# Death of John Wicks Heywood, M. D.

**Published:** 1854-06

**Authors:** B. Burwell, J. Root

**Affiliations:** President; Secretary


At the meeting of the members of the Medical Profession of this city, at the Clarendon, on Friday, 19th May instant, pursuant to notice, on the occasion of the death of John Wicks Heywood, M. D., B. Burwell was chosen President, and John Root, Secretary.

On motion of Dr. J. S. Trowbridge, it was *Resolved*, That a committee of five be appointed to prepare resolutions expressing the sense of the meeting

The following were appointed: Drs. Eastman, Wyckoff, Hawley, Johnson and Root.

On motion of Dr. White,

*Resolved*, That the members of the Medical Profession will attend the funeral of John Wicks Heywood, M. D., in a body, and wear the usual badge of mourning.

The Committee reported the following, which was unanimously adopted :

Whereas, We, the members of the Medical Profession in the City of Buffalo, have heard, with feelings of severest regret, of the sutten and unexpected death of John Wicks Heywood, M. D., on Wednesday, the 17th inst—

*Resolved*, That in the removal by death of our friend, we recognize the hand of an all-wise Providence, who, while He chastens and afflicts the living, sustains and supports those who trust in Him.

*Resolved*, That in the death of Dr. Heywood, the Medical Profession mourn the loss of one whose highly cultivated intellect, extensive acquirements, unsullied moral worth, and energy of character, gave token of distinction in the profession of his choice.

*Resolved*, That a copy of these resolutions, signed by the Chairman and and Secretary, be transmitted to the family of the deceased, with the assurance of our profound sympathy in their afflictions, and of earnest hope and prayer that the God of mercy, who chastens those whom He loves, will in due seasen pour into their hearts that full and abiding consolation which He alone can give.

*Resolved*, That a copy be also submitted to the *Buffalo Medical Journal.* and daily papers for publication. B. BURWELL, President.

J. Root, Secretary.

